# Identification of Transcriptional Signatures Associated with COPD in Immune and Epithelial–Stromal Cells Using Machine Learning Methods

**DOI:** 10.3390/life16101729

**Published:** 2026-10-15

**Authors:** Jingxin Ren, Jingjing Fan, Yusheng Bao, Wei Guo, Kaiyan Feng, Jerry Hu, Wenran Zhou, Deling Wang, Lin Lu, Tao Huang, Yu-Dong Cai

**Affiliations:** 1 School of Life Sciences, Shanghai University, Shanghai 200444, China; ssdrg@shu.edu.cn (J.R.); bys@shu.edu.cn (Y.B.); emilyzhou@shu.edu.cn (W.Z.); 2 Department of Radiotherapy, The Ninth Medical Center, Chinese PLA General Hospital, Beijing 100853, China; fj801207@163.com; 3 Shenzhen Institute of Advanced Technology, Chinese Academy of Sciences, Shenzhen 518055, China; gw_1992@sjtu.edu.cn; 4 Department of Computer Science, Guangdong AIB Polytechnic College, Guangzhou 510507, China; kyfeng@gdaib.edu.cn; 5 Department of Natural Sciences and Mathematics, College of Natural and Applied Sciences, Texas A&M University-Victoria, Victoria, TX 77901, USA; huj@tamuv.edu; 6 State Key Laboratory of Oncology in South China, Guangdong Provincial Clinical Research Center for Cancer, Sun Yat-Sen University Cancer Center, Guangzhou 510060, China; wangdl@sysucc.org.cn; 7 Department of Radiology, Memorial Sloan Kettering Cancer Center, New York, NY 10065, USA; 8 Bio-Med Big Data Center, CAS Key Laboratory of Computational Biology, Shanghai Institute of Nutrition and Health, University of Chinese Academy of Sciences, Chinese Academy of Sciences, Shanghai 200031, China; 9 Department of Artificial Intelligence and Digital Health, CAS Engineering Laboratory for Nutrition, Shanghai Institute of Nutrition and Health, University of Chinese Academy of Sciences, Chinese Academy of Sciences, Shanghai 200031, China

**Keywords:** chronic obstructive pulmonary disease, gene expression, machine learning, feature selection

## Abstract

In the context of chronic obstructive pulmonary disease (COPD), understanding gene expression patterns across various cell types is pivotal for disease comprehension, therapeutic development, and enhancement of patient prognosis. Nevertheless, the intricate interplay between immune and structural cells, along with their significance in COPD pathogenesis, remains only partially understood. This research employed multiple machine learning techniques to pinpoint marker genes exhibiting pronounced alterations between COPD patients and healthy individuals. We utilized six feature selection algorithms (LASSO, LightGBM, MCFS, RF, CATboost, and XGBoost) and three classification methods (DT, KNN, and RF) to analyze single-cell patient data. Cells were bifurcated into two subsets: immune cells and epithelial–stromal cells. Each cell, encompassing 21,237 genes, was labeled either as a healthy control or COPD. We identified a number of promising genes, such as FKBP5, which encodes a protein crucial for stress response regulation, and SCGB3A1, which is predominantly expressed in the respiratory tract and critical for regulating lung function. Both genes exhibit strong correlations with COPD. The study also underscored the potential roles of HLA-DRB1 and HSP90AA1 in COPD pathogenesis. Our findings underscore the unparalleled potential of machine learning in identifying genetic markers for intricate diseases like COPD, paving the way for targeted therapeutic advancements.

## 1. Introduction

Chronic obstructive pulmonary disease (COPD), a chronic and progressive lung disease, affects millions globally. This disease is characterized by a progressive airflow restriction that is not fully reversible, primarily induced by prolonged exposure to harmful particulates or gases. While tobacco smoke stands as the predominant risk factor, other elements such as environmental pollution, occupational exposures to dust and chemicals, and genetic predispositions also play significant roles in its onset [1]. The pathogenesis of COPD is multifaceted, encompassing a series of processes including persistent inflammation, oxidative stress, protease–antiprotease imbalance, and irregular tissue repair mechanisms [2].

The immune system is instrumental in driving the inflammatory process in COPD. Crucial immune cells, including neutrophils, macrophages, T lymphocytes, and B lymphocytes, mediate the inflammation of the respiratory tract and pulmonary tissues. Notably, neutrophils are the most abundant immune cells in the lungs of patients with COPD, discharging an array of harmful agents that impair lung tissues. Past research established that in COPD patients, neutrophils excrete elevated concentrations of reactive oxygen species and proteases, culminating in lung damage and inflammation [3]. Furthermore, macrophages are conspicuously present in COPD-afflicted lungs, where they spearhead the phagocytosis of bacteria and extraneous particles. A 2014 investigation delved into macrophages’ contribution to emphysema in COPD patients [4]. The findings revealed that macrophages produced higher levels of pro-inflammatory cytokines like IL-1β and TNF-α in COPD patients compared to their healthy counterparts. This study also identified macrophages’ role in the deterioration of alveolar structures in emphysema patients. Moreover, T and B lymphocytes contribute to pulmonary inflammation by synthesizing cytokines and antibodies, such as IL-17, IFN-γ, IL-6, and IL-8, which accentuate disease advancement [5,6].

In the context of COPD pathogenesis, structural cells like epithelial and stromal cells also play a role in the pathogenesis of COPD. The epithelial layer, acting as the primary defense against inhaled irritants, is integral for mucociliary clearance. The stroma, on the other hand, bestows structural integrity to the lungs and plays a role in tissue repair and remodeling [7]. Stromal cells, encompassing fibroblasts, smooth muscle cells, and endothelial cells, are implicated in COPD’s pathogenesis [8]. These cells synthesize extracellular matrix proteins, instigating airway remodeling and fibrosis, consequently exacerbating airflow limitations. Furthermore, stromal cells secrete chemokines and growth factors, recruiting and activating immune cells, thus escalating inflammation and tissue damage. Contemporary research accentuates the paramount interactions between immune cells and epithelial–stromal cells in COPD’s onset and progression. Reports indicate T lymphocytes’ interaction with fibroblasts and smooth muscle cells, promoting airway remodeling and fibrosis [9]. Analogously, B lymphocytes engage with epithelial cells, intensifying pulmonary inflammation [10]. These interplays between immune and structural cells are intricate, contingent on the specific cellular types and the particular cytokines and chemokines generated.

Utilizing single-cell sequencing data, our study employed a range of machine learning algorithms to discern genes and rules with marked differences between specific cell types (immune cells and epithelial–stromal cells) in COPD patients and healthy controls. These insights pave the way for a deeper understanding of COPD’s pathogenesis, bolstering early diagnosis, prognosis, tailored treatments, and drug development for the disease. The identified genes and rules hold potential as biomarkers for the early diagnosis and prognosis of COPD [11]. Such biomarkers could empower clinicians to pinpoint individuals at heightened risk of COPD, facilitating timely interventions and personalized treatment strategies. Additionally, by targeting differentially expressed genes and rules in COPD patients, researchers can craft drugs that address the disease’s fundamental mechanisms. Consequently, recognizing genes and rules with pronounced changes between specific cell types—namely, immune cells and epithelial–stromal cells—in COPD and healthy controls is pivotal for comprehending the disease, formulating efficacious therapies, and enhancing patient outcomes.

## 2. Materials and Methods

**Figure 1:**
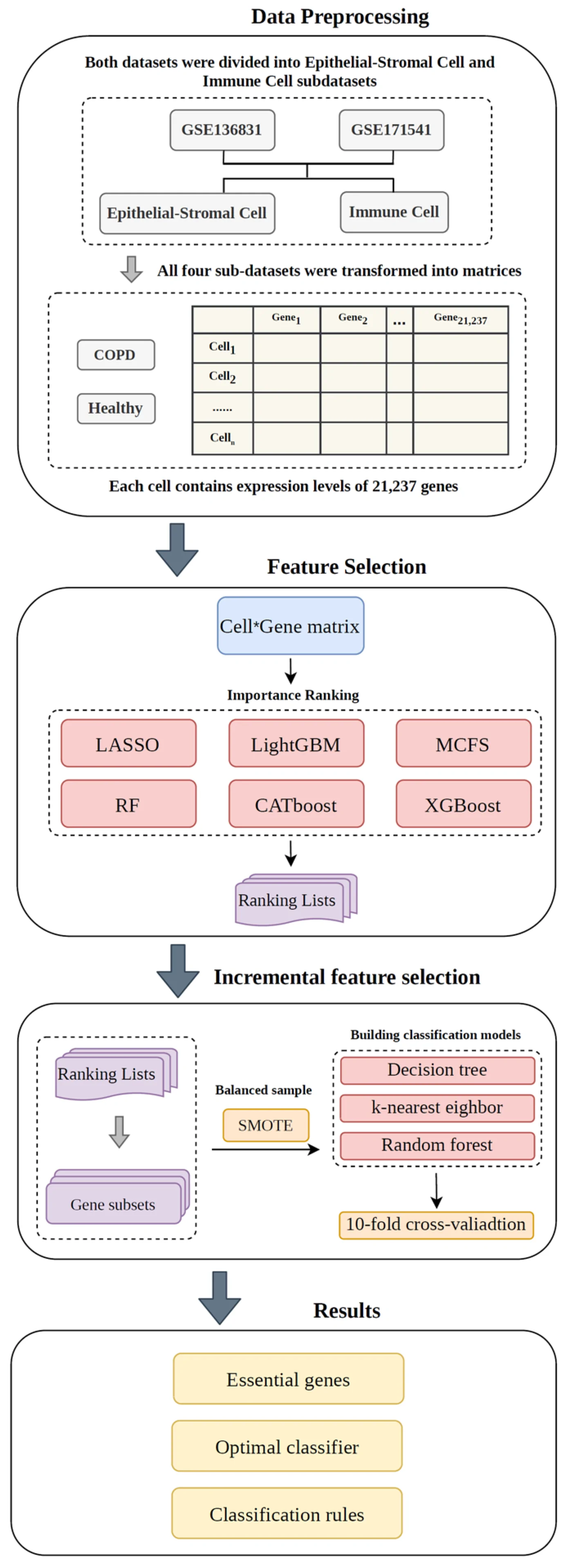
Flow chart of the entire analysis process. Two sets of RNA-seq data from COPD patients were analyzed using machine learning methods. Each data set was divided into two sub-datasets, epithelial–stromal cell and immune cell, and the cell samples included COPD and controls. Gene sets were evaluated for significance by six feature-ranking algorithms and passed to the incremental feature selection method. Effective prediction models and classification rules were constructed based on decision tree, random forest, and k-nearest neighbor algorithms.

We initially converted the datasets into cell–gene matrices, encompassing two cell types. Cells were classified by their corresponding COPD type, laying a structured foundation for ensuing procedures. We employed six feature-ranking algorithms to prioritize gene sets based on importance, obtaining six feature lists. Then, the incremental feature selection (IFS) [12] method was invoked to analyze each feature list. We constructed classification models using three classification algorithms, Decision Tree (DT) [13], Random Forest (RF) [14], and k-nearest neighbor (KNN) [14], on feature subsets containing some top features in the list. In this procedure, the Synthetic Minority Over-sampling Technique (SMOTE) [15] was used to balance the datasets, ensuring class size equilibrium. Employing 10-fold cross-validation, we gauged the models’ performance. Finally, we were able to construct prediction models with acceptable performance for different cell types and also identified a crucial set of genes that had significant predictive power. The entire procedure is illustrated in Figure 1.

### 2.1. Data

In this study, we utilized single-cell data from COPD patients sourced from Huang et al. [16] (with GEO database accession number GSE171541) and Adams et al. [17] (GEO database accession number GSE136831). The datasets were categorized based on cell type, resulting in two distinct sub-datasets for each: immune cells and epithelial–stromal cells. Within GSE171541, the immune cells encompassed B cells, dendritic cells, macrophages, mast cells, monocytes, natural killer cells, neutrophils, and T cells. The Epithelial–Stromal Cells included alveolar type 1 and 2 cells, ciliated cells, club cells, endothelial cells, proliferating cells, and stromal cells. For GSE136831, immune cells included Lymphoid and Myeloid categories, while epithelial–stromal cells included Endothelial, Epithelial, and Stromal. Each cell was designated as either COPD or healthy control. For our analysis, 21,237 genes present in cells across both datasets were chosen as features. The sample sizes for the four sub-datasets are detailed in Table 1. The GSE171541 cohort comprised nine donors, including three patients with COPD and six controls (three older and three younger controls), whereas the COPD-control portion of GSE136831 comprised 46 donors, including 18 patients with COPD and 28 control donors [16,17]. Disease labels assigned to individual cells were inherited from their corresponding donors. The aggregation into immune cell and epithelial–stromal cell subsets was intended to identify broad compartment-level transcriptional signatures rather than cell-subtype-specific effects.

### 2.2. Feature-Ranking Algorithms

To find genes in the gene set that are closely linked to COPD, we processed four sub-datasets using six feature-ranking algorithms, including Least Absolute Shrinkage and Selection Operator (LASSO) [18], Light Gradient Boosting Machine (LightGBM) [19], Monte Carlo Feature Selection (MCFS) [20], RF [14], Categorical Boosting (CATboost) [21], and Extreme Gradient Boosting (XGBoost) [22]. Each gene is assigned an importance score in this process, and the score implies the degree of association of the feature with the target variable. Based on the ranking results, the range where the key genes are located can be significantly narrowed down to inform the interpretation of the prediction results. These ranking methods have been widely used in existing studies [16,23,24,25]. The brief descriptions of these algorithms are provided in File S1.

### 2.3. Incremental Feature Selection

IFS [12] is a sequential method for selecting features in machine learning algorithms. It is typically applied to high-dimensional datasets where it is difficult to visualize the data or interpret the models. In IFS, features are added to the model incrementally, one by one, and the performance of the model is monitored at each step. The aim is to find the smallest subset of features that achieves near-maximum performance, simplifying the model, reducing overfitting, and improving interpretability. Here is how the process generally works: 1. Rank Features: First, the features are ranked according to some criteria. 2. Add Features Incrementally: Starting with the highest-ranked feature, features are added to the model one at a time in the order of their ranking. 3. Evaluate Model Performance: After each addition, the model is trained, and its performance is evaluated using a validation set or cross-validation. 4. Select Optimal Feature Subset: The process continues until all features are added or adding more features does not significantly improve the performance of the model. The subset of features that gives the best performance is selected as the optimal feature set.

### 2.4. Synthetic Minority Oversampling Technique

SMOTE is a popular technique for addressing class imbalance in machine learning datasets [15]. Class imbalance is a common problem in many real-world classification tasks, where the majority class (or classes) have many more instances than the minority class. This can lead to poor performance of the classifier, as it tends to be biased towards the majority class. SMOTE is an oversampling method that aims to balance the class distribution through the creation of synthetic examples rather than by oversampling with replacement. Here is how SMOTE works: 1. For a randomly chosen instance in the minority class, identify its K nearest neighbors. These neighbors will also belong to the minority class. 2. Select one of these neighbors randomly and connect this instance with the original one. 3. The synthetic instance is then placed somewhere along the line joining the two instances. The exact location is determined randomly, meaning that the synthetic instances are a kind of random linear interpolation between two existing instances. 4. Repeat this process until the desired class balance is achieved. SMOTE is effective for addressing class imbalance in most cases. However, when the dimension is high, SMOTE may induce geometric limitations and metric degradation, as distance metrics in SMOTE may lose contrast in high-dimensional spaces, causing linear interpolation to generate synthetic samples in empty regions of the data manifold and introducing severe noise.

In this study, SMOTE was used in the IFS procedure. Before using the top features in one list to construct prediction models, we adopted SMOTE to balance the sizes of all classes. When executing 10-fold cross-validation, SMOTE was only applied to the training set to avoid data leakage in each round of cross-validation.

### 2.5. Classification Algorithm

In this study, three classification algorithms are synthesized, including DT [13], KNN [26] and RF [14]. The description of RF can be found in File S1, and the descriptions of DT and KNN are as follows.

#### 2.5.1. Decision Tree

A DT [13] is a graphical representation of possible solutions to a decision, based on certain conditions. DTs are used widely in machine learning and artificial intelligence to solve both classification and regression problems. The structure of a DT is similar to a flowchart, with the ‘root’ at the top that branches off into some nodes, which can further branch into more nodes until a ‘leaf’ node is reached, indicating a decision. The root node is where the decision-making process begins. The decision node represents a test or decision on the dataset, the outcome of which splits the dataset into two or more subsets based on attribute selection measures such as Information Gain, Gain Ratio, or Gini Index. The branches are outcomes of the tests on each decision node. They connect the nodes and help to visualize the relationship between the decision nodes and the outcomes. Leaf nodes provide the final decision, and they represent the output or decision.

#### 2.5.2. k-Nearest Neighbors

KNN [26] is a type of instance-based learning or lazy learning algorithm, used for both regression and classification tasks. Its operation is quite simple to understand—the label of an unknown instance is determined by the labels of its nearest neighbors in the feature space. Here is a step-by-step breakdown of how the KNN algorithm works: 1. Choose the number K of neighbors: This is typically a small, odd integer to avoid ties. 2. Calculate the distance: The distance between the new point (whose classification is unknown) and all the other points in the dataset is calculated. 3. Find closest neighbors: Identify the ‘K’ points in the training data that are closest to the new point. 4. Vote for labels: All the ‘K’ points vote on the classification of the unknown point. The majority rule is typically used for voting. 5. Assign the class label: The new data point is assigned to the class that has the majority of votes.

### 2.6. Performance Evaluation

In this study, the performance of the classifier was evaluated using 10-fold cross-validation [27,28,29,30,31,32,33]. The F1 measure [34,35,36,37,38,39] is a measure of a model’s performance on binary classification tasks. The precision and recall are conceptually simpler, but they can be misleading if used alone. The F1 measure considers both the precision and the recall of the test to compute the score.

(1) $$\mathrm{Precision}=\frac{TP}{TP+FP},$$

(2) $$\mathrm{Recall}=\frac{TP}{TP+FN},$$

(3) $$F1\;\mathrm{measure}=\frac{2\times (\mathrm{Recall}\times \mathrm{Precision})}{\left(\mathrm{Recall}+\mathrm{Precision}\right)},$$

where TP is true positive, FP is false positive, and FN is false negative. Classifier performance increases with F1 measure. When computing the above metrics, healthy control cells were termed as positive samples, where COPD cells were regarded as negative samples. In this study, we used feature ranking algorithms to generate feature lists on each sub-dataset before executing 10-fold cross-validation. This operation introduced mild data leakage in cross-validation, which produced somewhat optimistic values for the aforementioned metrics.

## 3. Results

We subjected the 21,237 candidate genes to six feature-ranking algorithms, assigning each gene a score indicative of its association with COPD. Genes with notably high scores are posited to be instrumental in COPD’s development. For every sub-dataset, the results yielded six ranked lists, which are provided in Tables S1–S4.

### 3.1. Performance of Classification Models Generated Based on Four Sub-Datasets

Each list comprises a large number of features (genes). As important genes are generally not many, we only considered the top 1000 genes in each list. Furthermore, by setting the separation spacing at 5, 200 gene subsets were constructed from the top 1000 genes. On each gene set, classification models were constructed using the DT, KNN, and RF algorithms after the dataset was processed by SMOTE. The model’s performance was assessed through ten-fold cross-validation. In each round of cross-validation, SMOTE was only applied to the training set, as mentioned in Section 2.4. The IFS curves were plotted in Figures S1–S4. On these curves, the F1 measure is aligned to the *Y*-axis, while the number of features is plotted on the *X*-axis. Detailed performance metrics for all models can be found in Tables S5–S8.

For the epithelial–stromal cell sub-dataset from GSE136831, no single best classification model is consistently evident. However, both the KNN and RF models consistently outperform the DT model across all lists. This suggests that the KNN and RF algorithms are better suited for high-dimensional feature spaces in this sub-dataset. While no feature-ranking algorithm clearly outperforms the others, the performances of the top classification models across most lists are comparable. Notably, the KNN model applied to the LASSO list emerges as the top performer, achieving an F1 measure of 0.871 when utilizing 195 genes. The performance metrics for the top classification model for this sub-dataset are detailed in Table 2.

In the immune cell sub-dataset from GSE136831, the DT model’s performance lags behind that of the RF and KNN models. The KNN model consistently stands out across all six sorted lists. Specifically, the KNN model applied to the XGBoost list, utilizing 165 genes, achieves the highest F1 measure of 0.886. Importantly, while this F1 measure is marginally superior, this model requires the fewest genes among the lists, underscoring its efficiency. Table 3 presents the performance of the top classification model for this sub-dataset.

For the epithelial–stromal cell sub-dataset from GSE171541, the RF model consistently outpaces the other two models. Notably, the performance gap between the DT and KNN models is negligible for all lists except the LASSO list. The top-performing RF model across lists boasts an F1 measure exceeding 0.900. The peak performance is achieved with the LightGBM list, utilizing 105 genes and registering an F1 measure of 0.929. As this model uses the fewest genes among all lists, it excels in both performance and efficiency. Table 4 provides the performance metrics for the top classification model of this sub-dataset.

For the immune cell sub-dataset from GSE171541, akin to the previous GSE171541 sub-dataset, the RF model consistently shines. This indicates the RF model’s adaptability to the high-dimensional data of GSE171541. Among all lists, the pinnacle of performance is attained with the LightGBM list, using merely 70 genes and achieving an F1 measure of 0.947. Given its minimal gene requirement, this model stands out in terms of both performance and efficiency. The performance of the best classification model for this sub-dataset is elaborated in Table 5.

### 3.2. Patient-Level Test on Best Models

In Section 3.1, all tests were conducted at the cell level, suggesting cells from the same patient may occur in both training and test sets in each round of cross-validation. Here, we conducted a strict patient-level test on the best models of four sub-datasets. Patient-level expression profiles of four sub-datasets were generated by summing the raw UMI counts for each gene across all cells from the same patient. The aggregated counts were then normalized to counts per million (CPM), using the total UMI counts across all genes in the included cells as the library size, followed by a natural logarithmic transformation, log1p(CPM). Each patient was labeled as either healthy/control or COPD for subsequent machine-learning analysis. Based on the features in the best models, patients were also represented by these features. Each best model was evaluated on the corresponding patient-level sub-dataset by cross-validation. The accuracy (ACC), Matthews correlation coefficient (MCC), and F1 measure are listed in Table 6. These metrics were computed using healthy controls as positive samples and COPD patients as negative samples. It can be found that the performance on the three sub-datasets is quite high, with the F1 measure higher than 0.9. Compared with the performance under cell-level testing, listed in brackets in Table 6, the best models provided better performance on two sub-datasets of GSE136831. The performance on the immune cell sub-dataset of GSE171541 was slightly lower, whereas the performance on the epithelial–stromal cell sub-dataset of GSE171541 was not satisfactory, evidently lower than that under the cell-level test. Overall, the patient-level test results were close to the cell-level test results, implying the features/genes identified through the cell-level test were effective, which can also indicate differences between COPD and healthy controls at the patient level.

### 3.3. Key Genes Identified by Several Algorithms in Four Sub-Datasets

**Figure 2:**
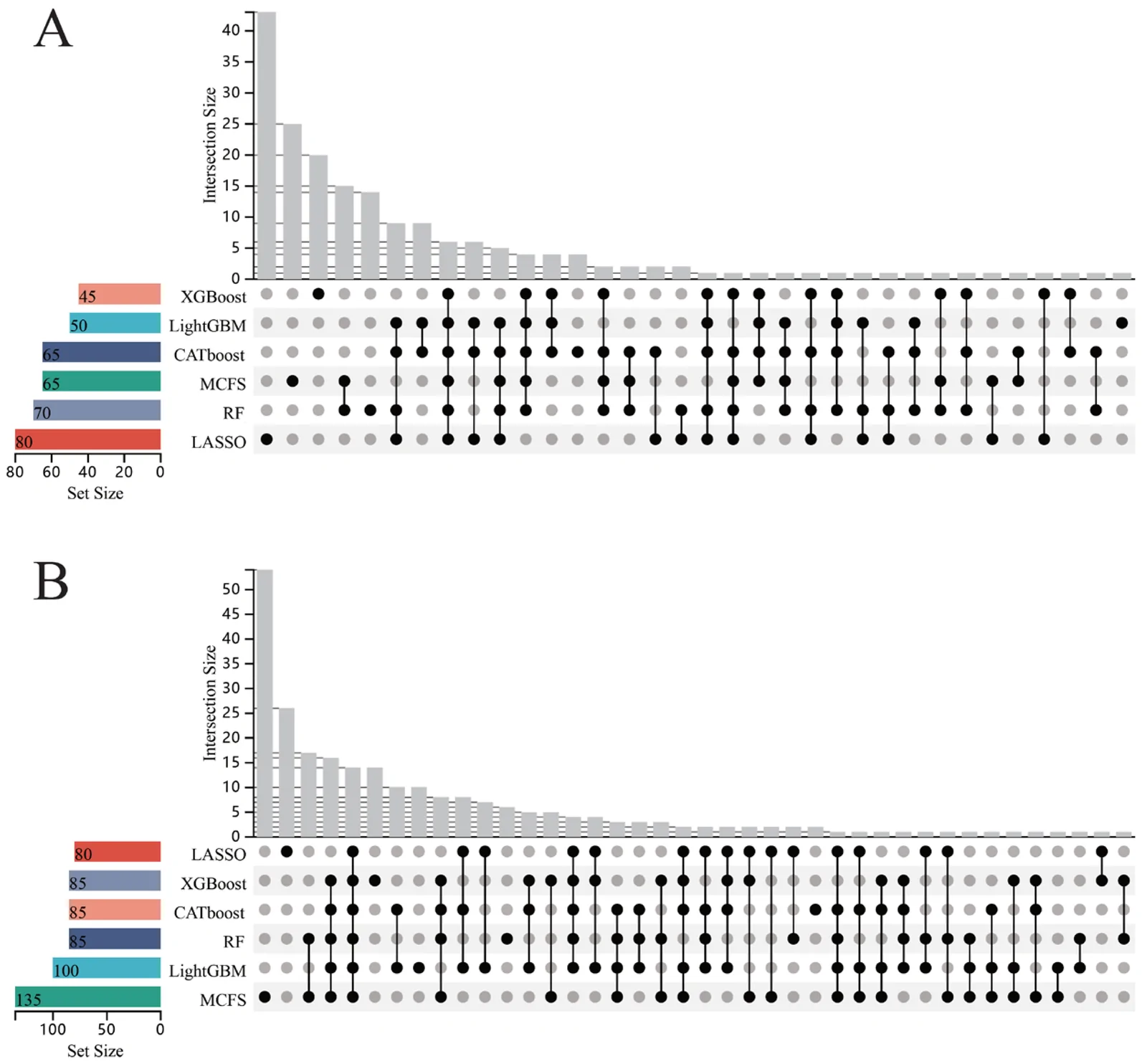
For GSE136831, upset graph of the essential features obtained using LASSO, LightGBM, MCFS, RF, CATBoost, and XGBoost. Venn diagrams are plotted based on the number of occurrences of gene signatures and the subsets in which they are located. The black dots represent the subsets where the features are located. (**A**) Venn diagram of epithelial–stromal cell sub-dataset. (**B**) Venn diagram of immune cell sub-dataset.

**Figure 3:**
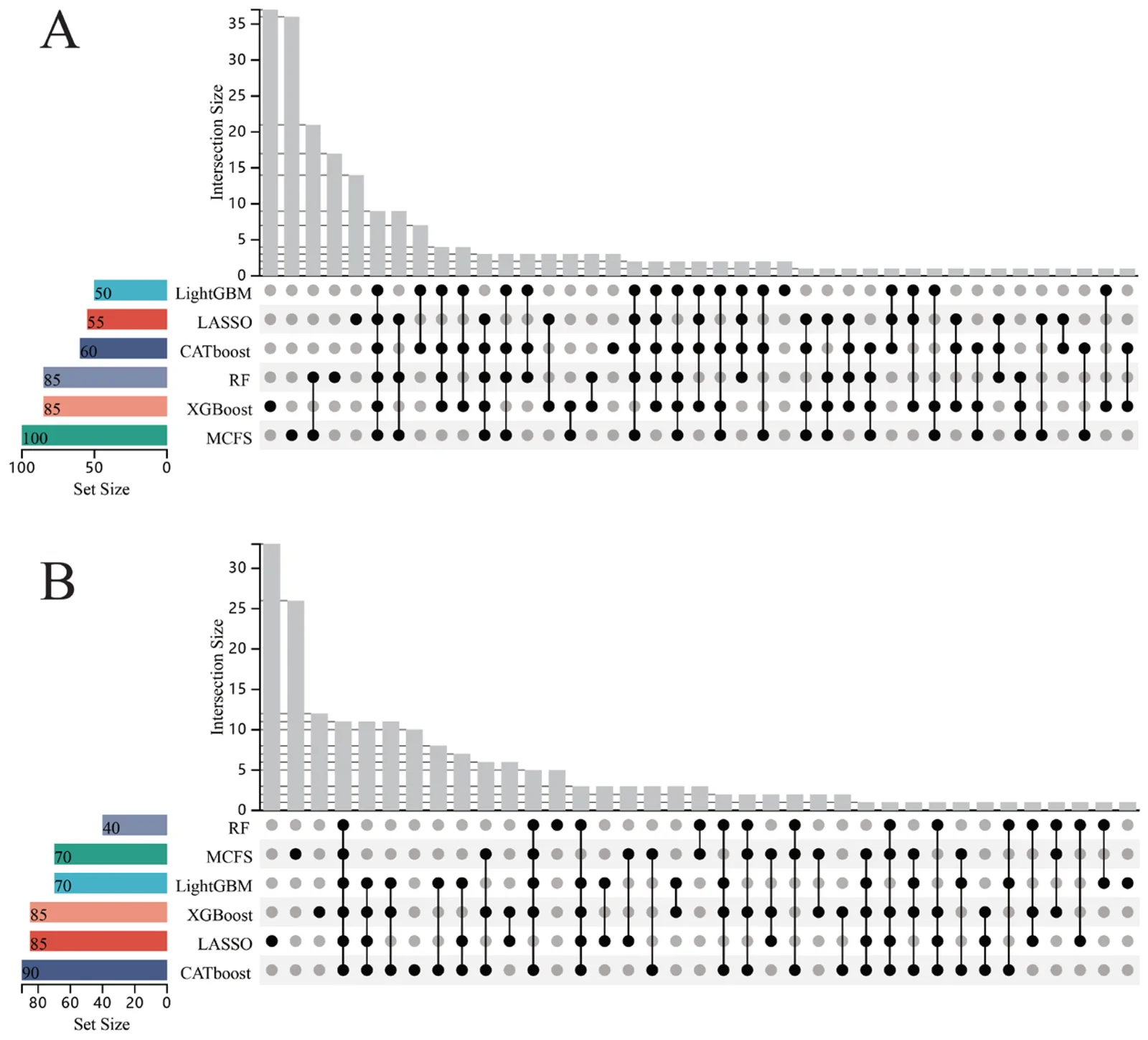
For GSE171541, an upset graph of essential features obtained using LASSO, LightGBM, MCFS, RF, CATBoost, and XGBoost. Venn diagrams are plotted based on the number of occurrences of gene signatures and the subsets in which they are located. The black dots represent the subsets where the features are located. (**A**) Venn diagram of epithelial–stromal cell sub-dataset. (**B**) Venn diagram of immune cell sub-dataset.

**Figure 4:**
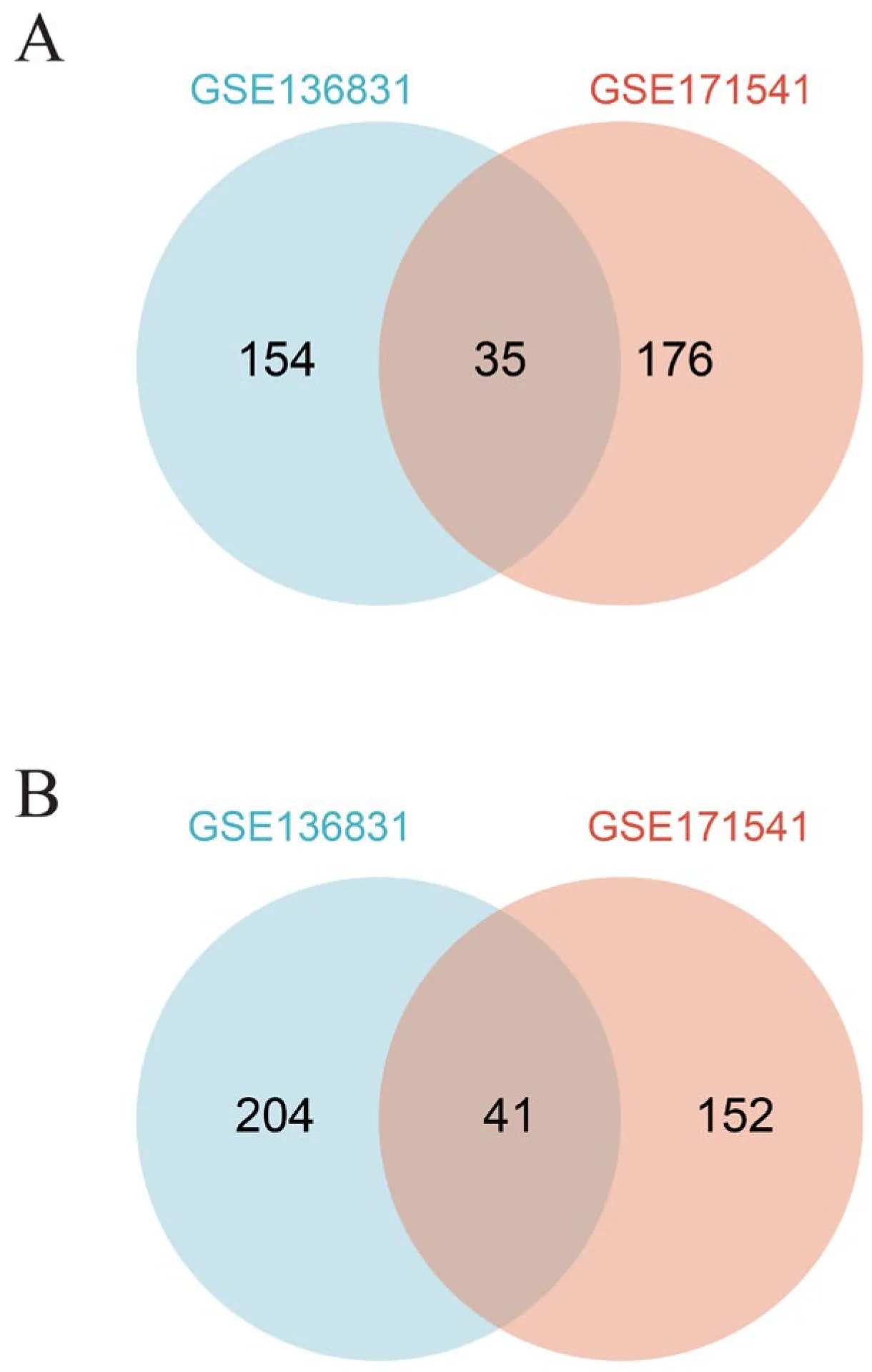
For the GSE136831 and GSE171541 datasets, intersection results for genes before the inflection point of the IFS curve. Overlapping circles indicate the essential genes shared by the two datasets. (**A**) Venn diagram of epithelial–stromal cell. (**B**) Venn diagram of immune cell.

Based on the IFS results, we identified the top-performing classification models across different lists for the four dataset subsets. Genes frequently deemed crucial by multiple methods were of particular interest. To pinpoint these common genes, we intersected the optimal gene subsets derived from various feature ranking algorithms. It is crucial to acknowledge that due to the voluminous number of genes in certain optimal gene subsets, redundant information is prevalent. Consequently, we opted for gene subsets at the IFS curve’s inflection points. By examining the IFS curves, we determined that models at these inflection points typically utilized fewer features, yet their F1 measures closely mirrored those at the curve’s peaks. The intersections were visualized using an upset graph (Figure 2 and Figure 3), with Table S9 detailing the intersection results. We further delve into frequently occurring genes in subsequent analyses. Each dataset highlights distinct key genes for both cell types. After merging the gene subsets from the inflection points across the six feature-ranking algorithms, we identified common key genes across both datasets (Figure 4). Specifically, 35 genes were shared for the epithelial–stromal cell type, and 41 for the immune cell type. Across diverse datasets and algorithms, these shared genes consistently emerged as pivotal in COPD’s pathogenesis.

### 3.4. Expression Trends of Key Genes in Different Datasets

**Figure 5:**
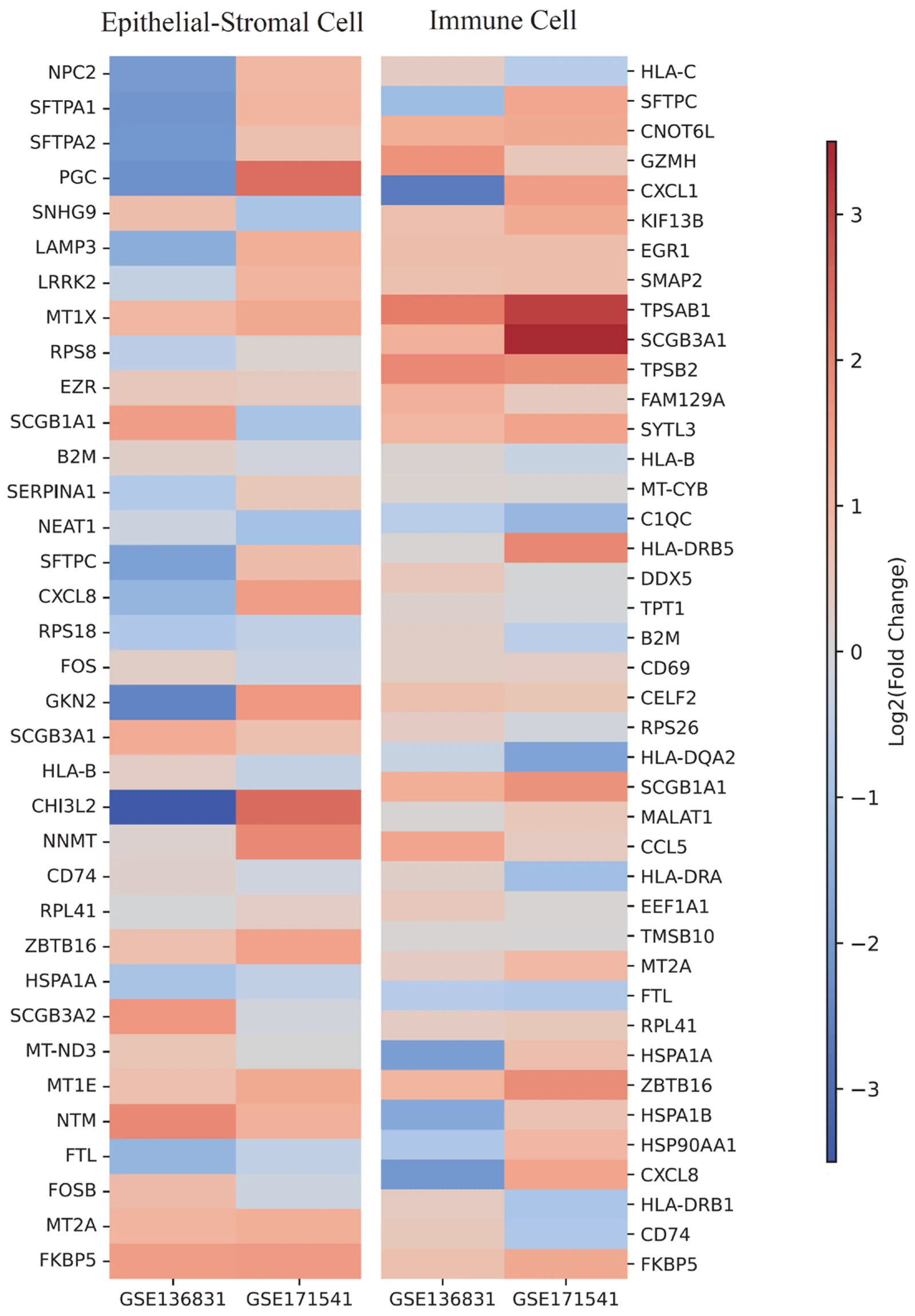
The expression heatmap of genes shared between two datasets based on Fold Change. The heat map contains two types of cells. Each row represents a gene, and each column represents the source of the cell sample. The shade of the color indicates the fold change in gene expression in COPD patients compared to controls.

For epithelial–stromal cells and immune cells, there were 35 and 41 shared genes in the two datasets, respectively. To illustrate the gene expression patterns, we computed the fold change in gene expression for each sample type within the datasets and depicted these trends using heatmaps (Figure 5). Within congruent cell types across the datasets, genes that ranked prominently on the list, such as *FKBP5*, EGR1, and *SCGB3A1*, consistently displayed an ascending trend. This indicates the successful identification of robust genetic markers across diverse datasets. Nonetheless, some genes exhibited divergent patterns. Given the variations in cell subtypes and their proportions between the datasets, the expression of certain genes might not consistently manifest across different subtypes.

### 3.5. Quantitative Classification Rules Based on DT Algorithm

The DT algorithm’s classification performance, when compared to RF and KNN, falls short across various datasets, but the performance of the DT model is more stable. Additionally, the well-documented predictive process of the DT model offers added value. By analyzing the DT model, we derived quantitative classification rules, representing the expression levels of key genes for distinct class labels. These rules aid in understanding the biological significance of key gene expression alterations in COPD. Tables S10–S13 detail the classification rules derived from different lists.

## 4. Discussion

### 4.1. The Discriminative Genes and Rules in Immune Cells

*FKBP5* emerges as a significant discriminative feature between the immune cells of COPD patients and healthy controls. This prominence is echoed in two single-cell sequencing profiles (GSE136831 and GSE171541) and is consistently identified by six distinct feature-ranking algorithms, namely, LASSO, LightGBM, MCFS, RF, CATboost, and XGBoost. The *FKBP5* gene encodes the FK506 binding protein 5, colloquially termed *FKBP5*1. This protein is instrumental in modulating the body’s stress response and is implicated in myriad physiological processes ranging from immune response and metabolism to neuroendocrine functionality. Critically, *FKBP5* regulates the immune chemokine interleukin-8 (IL-8), which predominantly orchestrates the recruitment and activation of neutrophils and other proinflammatory cells, thereby facilitating the immune response to infection and inflammation [40]. Anomalies in IL-8 expression and its functional dynamics correlate with several inflammatory and immune-mediated diseases, including COPD, asthma, rheumatoid arthritis, and even cancer. Zannas et al. increased the expression of *FKBP5* in human T cells (~3.7-fold) by transfection with *FKBP5* expression vectors, which leads to elevated levels of IL-8 secretion upon immune stimulation [41]. This highlights *FKBP5*’s potential in instigating the chemotaxis of proinflammatory cells. Meanwhile, conversely, melanoma cells, when subjected to *FKBP5* down-regulation, exhibit suppressed IL-8 production [42]. Furthermore, studies on immune cells have illuminated that heightened *FKBP5* levels intensify inflammation by strengthening the interactions of NF-κB (nuclear factor kappa-light-chain-enhancer of activated B cells) regulatory kinases [41]. Inhibiting *FKBP5*, either through strategic genetic deletion via CRISPR/Cas9 or through judicious pharmacological interventions, appears to counteract the influence of NF-κB. Since abnormal inflammatory response is one of the causes of COPD, aberrant expression of *FKBP5* in immune cells may result in immunological dysfunction through regulation of immune factors such as IL-8 and NF-κB and lead to the occurrence of COPD. Beyond COPD’s pathogenic underpinnings, *FKBP5*’s role in catalyzing corticosteroid insensitivity is well-established [43,44]. Recognized as a potent negative regulator of GR, *FKBP5* impacts the anti-inflammatory efficacy of corticosteroids. Intriguingly, experiments leveraging CRISPR-Cas9 to nullify *FKBP5* in the lung epithelial cell line A549 have spotlighted enhanced GR activity post Fluticasone Propionate (FP) treatment, hinting at *FKBP5*’s potential as a therapeutic target in COPD management [41]. In summation, FKBP5 is consistently and robustly associated with COPD. However, to encapsulate the intricacies of *FKBP5*’s role in COPD, especially its nexus with corticosteroid insensitivity, a more exhaustive exploration through rigorous studies and innovative experiments is paramount.

*CXCL8* emerges as a pivotal candidate feature, identified in GSE136831 by five feature-ranking algorithms and in GSE171541 by four feature-ranking algorithms. *CXCL8* is a pro-inflammatory cytokine that is primarily produced by activated monocytes and macrophages in response to various stimuli, including bacterial and viral infections, as well as inflammatory mediators such as tumor necrosis factor-alpha (TNF-alpha) and interleukin-1 beta (IL-1 beta). The primary function of *CXCL8* is to recruit and activate neutrophils, which are important in the innate immune response to infection and inflammation. Studies have shown that certain genes involved in inflammation and oxidative stress pathways are upregulated in COPD patients compared to healthy controls, including IL-8. Gilowska et al. have found that elevated levels of *CXCL8* are associated with an increase in neutrophil recruitment to the lungs, leading to chronic inflammation and exacerbation of COPD symptoms [45]. Furthermore, it has been observed that high levels of neutrophils in induced sputum correlate with a decline in lung function and increased disease severity. Thus, the combination of high *CXCL8* levels and an increased number of neutrophils in induced sputum may serve as a biomarker for predicting clinical outcomes in COPD patients. Therapeutic interventions aimed at reducing *CXCL8* levels may represent an effective strategy to mitigate inflammation and mucus hypersecretion in COPD. Notably, N-acetylcysteine (NAC), a glutathione precursor, has been demonstrated to be an inhibitor of *CXCL8*, *MUC5AC*, IL-6, and TNF, and its inhibitory effects on NF-κB factor and MAPK p38 phosphorylation have been shown to result in reduced clinical symptoms and frequency of COPD exacerbations, thus rendering it an attractive treatment option for this disease [45]. These insights underscore *CXCL8*’s pivotal role in the inflammatory processes driving COPD.

*HLA-DRB1* is consistently identified in GSE136831 by four feature-ranking algorithms and GSE171541 by six feature-ranking algorithms. This gene, which codes for the human leukocyte antigen DR beta chain, is situated on chromosome 6p21.3. The encoded protein is pivotal in the immune system’s recognition and response mechanisms against foreign entities like viruses and bacteria. Expressed on immune cells’ surfaces, including antigen-presenting cells such as dendritic cells, B cells, and macrophages, the *HLA-DRB1* protein is a major histocompatibility complex (MHC) class II molecule. Previous studies have linked certain HLA alleles to COPD [46]. Researchers conducted HLA medium-low resolution typing on 320 clinically stable COPD patients and 175 blood donor controls with the same ethnic and geographical origin. The results showed that while the HLA distribution was comparable between the COPD patients and controls, the HLA class II allele DRB1*14 could potentially play a role in the development of severe COPD with emphysema [47]. The *HLA-DRB1* protein binds to peptides derived from foreign substances and presents them to T cells, which then recognize and attack the foreign substance. The persistence of an enhanced inflammatory response mediated by T cells in the lungs of patients with COPD suggests an autoimmune reaction to a pan-pulmonary antigen that continues even after smoking cessation [48,49]. Therefore, we suggest *HLA-DRB1*’s potential involvement in COPD pathogenesis.

*HSP90AA1*, identified in both GSE136831 (by five feature-ranking algorithms) and GSE171541 (by six feature-ranking algorithms), encodes for the heat shock protein 90 alpha family class A member 1, commonly referred to as HSP90α. This protein is a molecular chaperone that plays a critical role in maintaining protein homeostasis by assisting in the proper folding, maturation, and stabilization of various client proteins. HSP90α is expressed in a wide variety of tissues and cell types, and its expression levels can be induced by various stressors, including heat, oxidative stress, and hypoxia. *HSP90AA1* is a key mediator of the occurrence of squamous cell lung carcinoma (SCC) caused by COPD, according to the published report [50]. A Network Analysis of microarray gene expression profiles highlighted the differential expression of *HSP90AA1* in SCC cases with and without COPD, underscoring its potential as a pivotal biomarker in COPD-induced SCC. Interestingly, SCC patients with lower *HSP90AA1* gene expression were found to have reduced overall survival rates compared to their counterparts with elevated gene expression. This necessitates further exploration into HSP90α’s role in COPD-induced SCC and the potential development of effective HSP90α inhibitors for clinical application.

Furthermore, our analysis also identified some genes that did not have strong computational support. For example, *EGR1* was identified in GSE136831 by one feature-ranking algorithm and in GSE171541 by three. *EGR1*, or Early Growth Response 1, is a zinc finger transcription factor that regulates gene expression by binding specific DNA sequences, contingent upon context. Recognized as a primary response gene, *EGR1* swiftly responds to extracellular triggers, orchestrating a gamut of physiological undertakings from cell growth to survival. Additionally, *EGR1*’s role in modulating immune responses to infections and inflammation has been documented. Immunohistochemical techniques have detected *EGR1* across diverse lung cell types, ranging from alveolar epithelial cells to inflammatory cells [51]. *EGR1* has been identified as a critical transcription factor for TNF-α by means of its binding to the TNF-α promoter [52]. TNF-α plays a pivotal role in the acute response to smoke and, in conjunction with other cytokines, may activate both inflammatory and structural cells, thereby inducing the production of matrix metalloproteinases and facilitating matrix breakdown, which represents one of the primary hallmarks of emphysema [51]. Moreover, EGR1 is dysregulated in COPD-patient lungs [53]. Cigarette smoke exposure induces EGR1, modulates MMP-2/MMP-9 activity, and may drive COPD-associated lung remodelling. Therefore, *EGR1* has been shown to be necessary for the development of normal immune responses, and its dysfunction has been linked to various immune-related disorders, including autoimmune diseases, immunodeficiency disorders, and immune dysregulation like COPD.

### 4.2. The Discriminative Genes and Rules in Epithelial–Stromal Cells

Both *SCGB3A1* and its homolog, *SCGB3A2*, are repeatedly identified across datasets: *SCGB3A1* in GSE136831 (by three feature-ranking algorithms) and GSE171541 (by four feature-ranking algorithms), and *SCGB3A2* in GSE136831 (by four feature-ranking algorithms) and GSE171541 (by five feature-ranking algorithms). These genes belong to the secret globin (SCGB) protein family, which is evolutionarily conserved among mammals. *SCGB3A1*, also known as HIN-1 (high in normal-1) and UGRP2 (uteroglobin-related protein 2), is primarily expressed in the respiratory tract, including the bronchi, bronchioles, and alveoli, where it has been shown to play a crucial role in modulating lung function and conferring protection against pulmonary diseases. *SCGB3A1* has also been identified as a potential biomarker for lung cancer and other respiratory diseases. *SCGB3A2*, also known as UGRP1, is expressed in the mammary gland, salivary glands, prostate, and lung. In a study by Hu et al. [54], the researchers utilized single-cell RNA sequencing (scRNA-seq) to investigate the expression patterns of various genes in distal human lung epithelial cells isolated from explanted lung tissues of patients with end-stage COPD and age-matched healthy donors. The results of the study showed that the COPD patients had an aberrant alveolar type II (ATII) cell subpopulation with elevated levels of *SCGB3A2* and *SCGB3A1* transcripts, compared to the healthy controls. This suggested that *SCGB3A2* and *SCGB3A1* may play a role in the impaired alveolar tissue regeneration observed in COPD. Overall, this study provides new insights into the pathogenesis of COPD and highlights the potential utility of scRNA-seq for identifying novel therapeutic targets. By identifying potential targets such as *SCGB3A2* and *SCGB3A1*, researchers may be able to develop new treatments that could improve alveolar tissue regeneration and function in COPD patients.

*HSPA1A* is both identified in GSE136831 by four feature-ranking algorithms and in GSE171541 by three feature-ranking algorithms. *HSPA1A* is a member of the heat shock protein 70 (HSP70) family, which is known to play a crucial role in cellular stress response and protein homeostasis. Specifically, *HSPA1A* is a stress-inducible molecular chaperone that binds to and stabilizes misfolded or denatured proteins, preventing their aggregation and facilitating their proper folding and refolding, which has been implicated in various physiological and pathological processes, including inflammation, immune response, carcinogenesis, and neurodegeneration. Several studies have established *HSPA1A*’s close association with COPD. For instance, Hacker et al. reported that *HSPA1A* levels were significantly higher in the serum of COPD patients compared to healthy controls [55]. Furthermore, the levels of *HSPA1A* correlated with disease severity and were particularly elevated in patients with severe COPD. Similarly, Dong et al. [56] investigated the association between *HSPA1A* expression and COPD severity and smoking status. They measured the expression of *HSPA1A* in bronchial biopsy samples from COPD patients and healthy controls, and found that *HSPA1A* expression was significantly higher in COPD patients compared to healthy controls. Moreover, they found that *HSPA1A* expression was strongly correlated with disease severity, as measured by lung function tests, and that expression was higher in current smokers compared to former smokers and non-smokers, suggesting that *HSPA1A* could also serve as a biomarker of smoking-induced inflammation in COPD. In the latest research, *HSPA1A* showed a significant association with COPD in a case–control study comprising 425 COPD patients and 457 controls with *p* = 0.006 [57]. In essence, *HSPA1A* emerges as a potential marker for immune activation and cellular damage in COPD, holding promise for monitoring disease progression and therapeutic responses.

The *MUC5AC* gene, pinpointed in GSE171541 by five feature-ranking algorithms, encodes the mucin protein *MUC5AC*. This protein is abundantly found in the respiratory tract and other mucosal tissues. Located on chromosome 11p15.5, the gene spans approximately 110 kb across 60 exons. *MUC5AC* is predominantly secreted by goblet cells in the respiratory tract’s surface epithelium and stands as a key component of the mucus gel layer lining the airway surface. This protein plays multifaceted roles in airway biology: from maintaining airway surface hydration and controlling mucociliary clearance to steering both innate and adaptive immune responses. *MUC5AC* gene has been shown to be one of the two major mucin genes that are upregulated by the *CXCL8* chemokine, alongside MUC5B, leading to excessive mucus production in patients with COPD. This overproduction of mucus can increase the frequency of COPD exacerbations and the risk of bacterial infections in affected individuals [45]. *MUC5AC* gene was found to be more reliably associated with manifestations of COPD compared to the MUC5B gene, based on a study involving 331 participants, including healthy never-smokers, at-risk ever-smokers, and ever-smokers with COPD [58]. Increased concentrations of *MUC5AC* were linked to decreased lung function, increased frequency of exacerbations, and higher COPD assessment scores. Additionally, *MUC5AC* concentrations were more reactive to cigarette smoke exposure compared to MUC5B concentrations. Longitudinal data from 3-year follow-up visits showed that high baseline *MUC5AC* concentration was a significant predictor of exacerbations and decline in lung function. Current smokers in the at-risk group had raised *MUC5AC* concentrations and decreased lung function over time, while former smokers in the at-risk group had normal *MUC5AC* concentrations and preserved lung function. Hence, a deeper understanding of *MUC5AC*’s regulation and function could offer invaluable insights into COPD’s pathogenesis and potential treatments.

**Figure 6:**
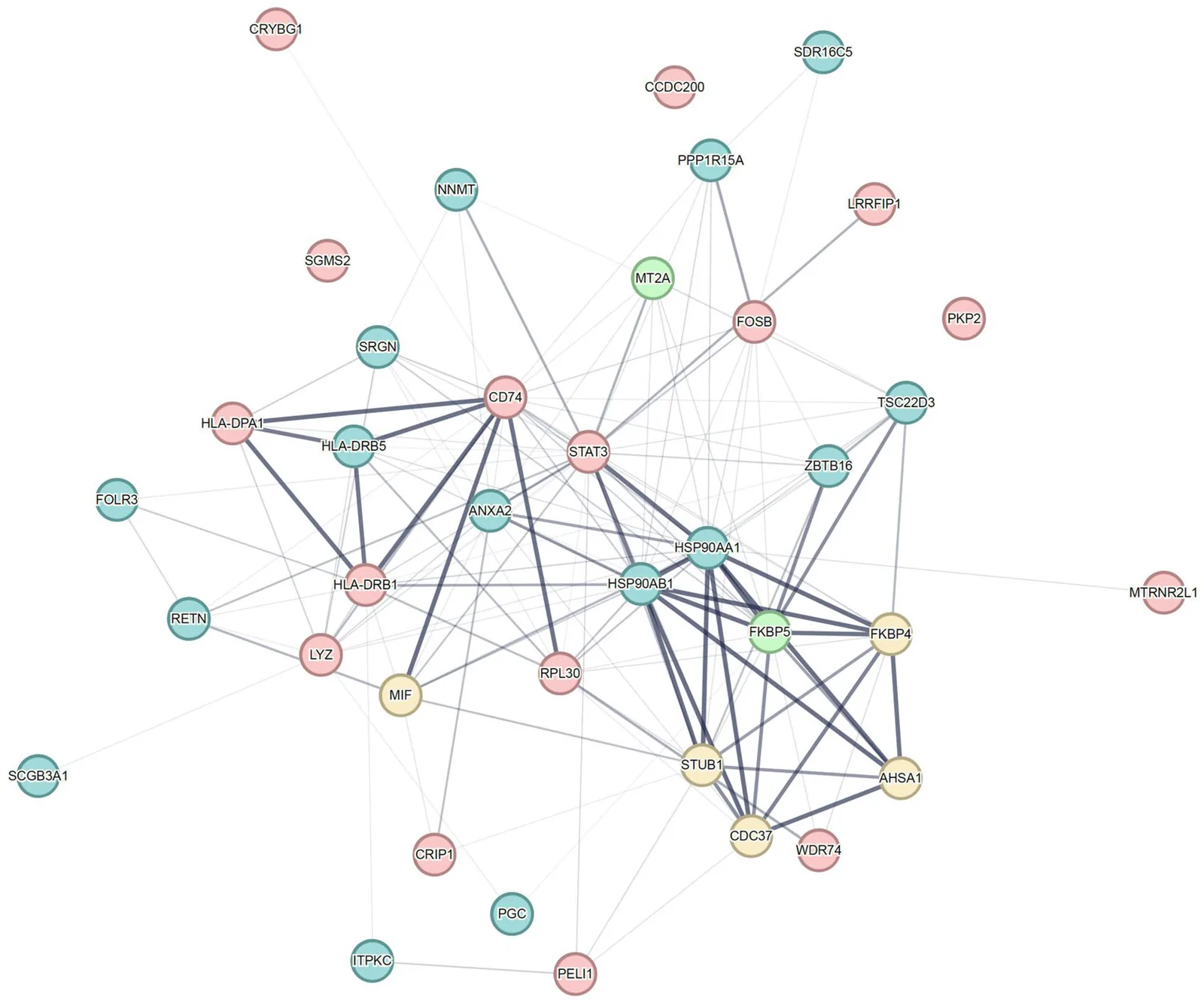
Network diagram of interrelationships of TOP proteins from the two datasets. The PPI network shows the association of proteins associated with COPD. This includes proteins encoded by genes screened by all algorithms in GSE136831 (red circles) and GSE171541 (blue circles). Green circles are proteins that appear in both datasets. Yellow circles are proteins that are highly correlated with our results. The lines between the circles represent interactions, and the thickness of the lines indicates the strength of the interaction. While this network simplifies complex biological processes, it visually highlights the major differential expression and its links between COPD patients and healthy populations.

Figure 6 offers a visual representation of the intricate relationships between the crucial gene products highlighted in this section. Notably, *FKBP5* and *HSP90AA1*, which were discussed in detail, exhibit extensive interactions with other proteins. The primary proteins identified in both datasets are intricately interconnected. Through this illustration, we were able to elucidate the complex interplay between essential genes linked to COPD and uncover several new proteins closely associated with our screened proteins. This visual aid is instrumental in pinpointing central proteins and identifying specific functional pathways.

### 4.3. Limitations

This study integrated single-cell transcriptomic datasets (GSE136831 and GSE171541) from different platforms and experimental conditions. Despite undergoing unified preprocessing and standardization, inherent heterogeneity persists across datasets in terms of cell classification, sample origin, sequencing depth, and experimental batches. This heterogeneity may lead to inconsistent gene expression trends extracted from different datasets. For example, certain genes may show an upregulation trend in one dataset but fail to exhibit the same pattern in another. Furthermore, variations in cell subtype definitions and proportions across studies may influence the overall gene expression landscape. Therefore, when interpreting shared genes across datasets, careful consideration must be given to the potential impact of these technical variations and biological heterogeneity on the reproducibility and generalizability of results. Future studies could further validate the stable role of these genes in COPD through larger-scale, multi-center integration of single-cell data combined with experimental validation.

Pooling heterogeneous cell types into the two broad immune cell and epithelial–stromal cell subsets introduces an additional limitation. Disease-associated differences in cell-subtype proportions may contribute to classifier performance and feature ranking, such that some selected genes may represent cell-lineage markers rather than within-cell-type COPD-associated transcriptional changes. For example, enrichment of a particular immune-cell subtype in COPD samples could increase the apparent predictive importance of that subtype’s baseline markers. Accordingly, the identified genes should be interpreted as candidate compartment-level signatures. Future analyses should quantify cell-type composition by donor and disease group and assess robustness using cell-type-specific, composition-balanced, or donor-level pseudobulk analyses.

The machine learning methods were applied to four cell-level datasets for extracting essential genes of COPD. The patient-level test in Section 3.2 was only applied to the best models that were built through cell-level testing. This test is not sufficient, as it used the genes selected from the same donors at cell-level evaluation and the sub-datasets of GSE171541 had only nine donors. Extensive patient-level tests are still needed.

Independent cross-dataset validation is now a necessary test process. This study also tried to conduct this test by using GSE136831 as the training cohort and GSE171541 as the independent testing cohort. However, the predictive performance was substantially reduced, which may be attributable to several factors, including differences in sequencing platforms, library preparation protocols, patient characteristics, and the limited samples in sub-datasets of GSE171541 (only nine donors). Therefore, the observed reduction in performance may reflect substantial technical and biological distribution shifts rather than a lack of disease-associated signal.

It is important to note that this study is computational and predictive in nature. The identified key genes and classification rules are based on machine learning models analyzing existing public datasets. Although we employed multiple feature selection methods, cross-validation, and parallel intersection analysis across two independent datasets to enhance result reliability, these findings require functional validation through subsequent experiments. For instance, gene knockout experiments, overexpression studies, protein interaction assays, and clinical sample validation will help elucidate the specific mechanisms and pathological roles of these genes in COPD pathogenesis. We encourage future studies to conduct in-depth functional exploration and translational medical evaluations of the candidate genes proposed in this study (such as FKBP5, SCGB3A1, HLA-DRB1, etc.) using in vitro cell models, animal experiments, or clinical cohorts. This will advance their practical application in early COPD diagnosis, prognosis assessment, and targeted therapy.

## 5. Conclusions

In this study, utilizing single-cell sequencing data, we discerned discriminative genes and patterns distinguishing COPD patients from healthy controls by employing a variety of machine learning algorithms. Our findings shed light on the intricate mechanisms underlying COPD, paving the way for the identification of potential therapeutic targets and the formulation of more effective treatment strategies. Our emphasis on immune and stromal cells underscores their potential as promising avenues for novel COPD therapies. For instance, interventions targeting neutrophils or macrophages could alleviate lung inflammation, thus decelerating COPD progression. Similarly, targeting fibroblasts or smooth muscle cells might curtail airway remodeling, leading to enhanced lung function. Given the cell-level validation and broad cellular aggregation used here, these findings represent hypothesis-generating compartment-level signatures and require donor-level and cell-type-specific validation before clinical application as patient-level biomarkers.

## Figures and Tables

**Table 1:** Number of cell samples included in the four sub-datasets.

Sub-Dataset	Class	Sample Size
GSE136831: Epithelial–Stromal Cell	Health Control	8135
	COPD	5236
GSE136831: Immune Cell	Health Control	87,168
	COPD	62,707
GSE171541: Epithelial–Stromal Cell	Health Control	12,762
	COPD	7346
GSE171541: Immune Cell	Health Control	32,166
	COPD	14,336

**Table 2:** For the epithelial–stromal cell sub-dataset of GSE136831, the performance of the best prediction models constructed from six lists when using the best feature subset and the inflection point subset.

Feature-Ranking Algorithms	Number of Features	F1 Measure	MCC	ACC
LASSO	80 *	0.861	0.647	0.831
	195 **	0.871	0.674	0.844
LightGBM	50 *	0.861	0.670	0.838
	170 **	0.869	0.698	0.849
MCFS	65 *	0.821	0.560	0.787
	330 **	0.842	0.607	0.811
RF	70 *	0.835	0.595	0.804
	310 **	0.844	0.614	0.814
CATboost	65 *	0.851	0.638	0.824
	385 **	0.866	0.667	0.840
XGBoost	45 *	0.841	0.610	0.811
	150 **	0.862	0.659	0.836

Those with * markers are the number of features at the inflection points of the IFS curve, and those with ** markers are the number of features in the best feature subset.

**Table 3:** For the Immune Cell sub-dataset of GSE136831, the performance of the best prediction models constructed from six lists when using the best feature subset and the inflection point subset.

Feature-Ranking Algorithms	Number of Features	F1 Measure	MCC	ACC
LASSO	80 *	0.871	0.686	0.848
	170 **	0.883	0.714	0.862
LightGBM	100 *	0.870	0.702	0.853
	215 **	0.884	0.738	0.870
MCFS	135 *	0.852	0.677	0.837
	685 **	0.874	0.730	0.862
RF	85 *	0.863	0.692	0.847
	250 **	0.881	0.734	0.867
CATboost	85 *	0.860	0.682	0.843
	175 **	0.883	0.735	0.868
XGBoost	85 *	0.862	0.691	0.846
	165 **	0.886	0.744	0.872

Those with * markers are the number of features at the inflection points of the IFS curve, and those with ** markers are the number of features in the best feature subset.

**Table 4:** For the epithelial–stromal cell sub-dataset of GSE171541, the performance of the best prediction models constructed from six lists when using the best feature subset and the inflection point subset.

Feature-Ranking Algorithms	Number of Features	F1 Measure	MCC	ACC
LASSO	55 *	0.901	0.729	0.874
	140 **	0.908	0.742	0.881
LightGBM	50 *	0.923	0.784	0.900
	105 **	0.929	0.802	0.909
MCFS	100 *	0.901	0.726	0.873
	485 **	0.914	0.760	0.890
RF	85 *	0.910	0.752	0.886
	320 **	0.919	0.772	0.895
CATboost	60 *	0.921	0.780	0.898
	320 **	0.928	0.798	0.907
XGBoost	85 *	0.921	0.781	0.899
	295 **	0.925	0.790	0.903

Those with * markers are the number of features at the inflection points of the IFS curve, and those with ** markers are the number of features in the best feature subset.

**Table 5:** For the immune cell sub-dataset of GSE171541, the performance of the best prediction models constructed from six lists when using the best feature subset and the inflection point subset.

Feature-Ranking Algorithms	Number of Features	F1 Measure	MCC	ACC
LASSO	85 **	0.930	0.774	0.903
LightGBM	70 **	0.947	0.830	0.927
MCFS	70 *	0.921	0.748	0.891
	480 **	0.928	0.767	0.900
RF	40 *	0.932	0.780	0.906
	145 **	0.938	0.802	0.915
CATboost	90 **	0.947	0.829	0.927
XGBoost	85 **	0.945	0.823	0.924

Those with * markers are the number of features at the inflection points of the IFS curve, and those with ** markers are the number of features in the best feature subset.

**Table 6:** Performance of the best models on patient-level sub-datasets ^$^.

Dataset	F1 Measure	MCC	ACC
GSE136831: Epithelial–Stromal Cell	0.947 (0.871)	0.863 (0.674)	0.935 (0.844)
GSE136831: Immune Cell	0.906 (0.886)	0.785 (0.744)	0.891 (0.872)
GSE171541: Epithelial–Stromal Cell	0.727 (0.929)	0.316 (0.802)	0.667 (0.909)
GSE171541: Immune Cell	0.909 (0.947)	0.791 (0.830)	0.889 (0.927)

^$^: Numbers in brackets are the metrics under the cell-level tests.

## Data Availability

The data analyzed in this study are available at the Gene Expression Omnibus database (https://www.ncbi.nlm.nih.gov/geo/, accessed on 10 January 2023). The analyzed results are contained within the article or Supplementary Materials.

## Supplementary Materials

The following supporting information can be downloaded at: https://www.mdpi.com/article/10.3390/life16101729/s1. File S1: Descriptions on feature-ranking algorithms. Figure S1: IFS curves on the Epithelial–Stromal Cell sub-dataset of GSE136831. Classification models were constructed using three algorithms. The F1 measure is set on the *Y*-axis and the number of features is set on the *X*-axis. (A) IFS curves based on LASSO. (B) IFS curves on LightGBM. (C) IFS curves based on MCFS. (D) IFS curves based on RF. (E) IFS curves based on CATboost. (F) IFS curves based on XGBoost. Figure S2: IFS curves based on the Immune Cell sub-dataset of GSE136831. Classification models were constructed using three algorithms. The F1 measure is set on the *Y*-axis and the number of features is set on the *X*-axis. (A) IFS curves based on LASSO. (B) IFS curves based on LightGBM. (C) IFS curves based on MCFS. (D) IFS curves based on RF. (E) IFS curves based on CATboost. (F) IFS curves based on XGBoost. Figure S3: IFS curves on the Epithelial–Stromal Cell sub-dataset of GSE171541. Classification models were constructed using three algorithms. The F1 measure is set on the *Y*-axis and the number of features is set on the *X*-axis. (A) IFS curves based on LASSO. (B) IFS curves based on LightGBM. (C) IFS curves based on MCFS. (D) IFS curves based on RF. (E) IFS curves based on CATboost. (F) IFS curves based on XGBoost. Figure S4: IFS curves on the Immune Cell sub-dataset of GSE171541. Classification models were constructed using three algorithms. The F1 measure is plotted on the *Y*-axis and the number of features is plotted on the *X*-axis. (A) IFS curves based on LASSO. (B) IFS curves based on LightGBM. (C) IFS curves based on MCFS. (D) IFS curves based on RF. (E) IFS curves based on CATboost. (F) IFS curves based on XGBoost. Table S1: For the Epithelial–Stromal Cell sub-dataset of GSE136831, feature ranking results obtained using LASSO, LightGBM, MCFS, RF, CATboost and XGBoost. Table S2: For the Immune Cell sub-dataset of GSE136831, feature ranking results obtained using LASSO, LightGBM, MCFS, RF, CATboost and XGBoost. Table S3: For the Epithelial–Stromal Cell sub-dataset of GSE171541, feature ranking results obtained using LASSO, LightGBM, MCFS, RF, CATboost and XGBoost. Table S4: For the Immune Cell sub-dataset of GSE171541, feature ranking results obtained using LASSO, LightGBM, MCFS, RF, CATboost and XGBoost. Table S5: Performance of three classifiers constructed using the IFS method on gene lists from the Epithelial–Stromal Cell sub-dataset of GSE136831. Table S6: Performance of three classifiers constructed using the IFS method on gene lists from the Immune Cell sub-dataset of GSE136831. Table S7: Performance of three classifiers constructed using the IFS method on gene lists from the Epithelial–Stromal Cell sub-dataset of GSE171541. Table S8: Performance of three classifiers constructed using the IFS method on gene lists from the Immune Cell sub-dataset of GSE171541. Table S9: For the four sub-datasets, the intersection essential features identified by LASSO, LightGBM, MCFS, RF, CATboost, XGBoost and RF. The features identified by 6, 5, 4, 3, 2 and 1 algorithm are shown. Table S10: For the Epithelial–Stromal Cell sub-dataset of GSE136831, the classification rules generated by the best DT classifier. Table S11: For the Immune Cell sub-dataset of GSE136831, the classification rules generated by the best DT classifier. Table S12: For the Epithelial–Stromal Cell sub-dataset of GSE171541, the classification rules generated by the best DT classifier. Table S13: For the Immune Cell sub-dataset of GSE171541, the classification rules generated by the best DT classifier.

## Abbreviations

The following abbreviations are used in this manuscript:

COPD	Chronic obstructive pulmonary disease
SMOTE	Synthetic minority over-sampling technique
IFS	Incremental feature selection
DT	Decision tree
RF	Random forest
LASSO	Least absolute shrinkage and selection operator
LightGBM	Light gradient boosting machine
MCFS	Monte Carlo feature selection
CATboost	Categorical boosting
XGBoost	Extreme gradient boosting
KNN	k-nearest neighbor
CPM	Counts per million
ACC	Accuracy
MCC	Matthews correlation coefficient
