# Supplementary material for: Identification of Transcriptional Signatures Associated with COPD in Immune and Epithelial–Stromal Cells Using Machine Learning Methods
Source: Life (Basel). 2026 Oct 15;16(10):1729. doi: 10.3390/life16101729 (PMC13706307; doi:10.3390/life16101729)
Supplement: Supplementary file 1 [file life-16-01729-s001.zip › File S1.pdf]

File S1. Descriptions on feature-ranking algorithms.

2.2.1 Least Absolute Shrinkage and Selection Operator (LASSO)

LASSO [1] is a popular method for regression analysis, introduced by Robert Tibshirani in 1996. LASSO works by adding a penalty term to the standard regression objective function, forcing the sum of the absolute values of the model's coefficients to be less than a specified constant (the regularization parameter, denoted as λ). This constraint encourages some of the coefficients to be exactly zero, effectively performing feature selection by shrinking the less important features' coefficients to zero. The result is a sparse model with only the most important features retained, making it easier to interpret and reducing the risk of overfitting. The feature importance by LASSO can be determined through the magnitude of the non-zero coefficients in the resulting model. Features with non-zero coefficients are considered important, as they have contributed significantly to the model's predictions. And the greater the magnitude of a feature, the more important the feature is considered.

2.2.2 Light Gradient Boosting Machine (LightGBM)

LightGBM [2] is a gradient boosting framework developed by Microsoft that uses decision tree-based learning algorithms. It's designed to be quick and efficient, and it stands out because of its capacity to handle large-sized data, ability to run on distributed and GPU-based systems, and its effectiveness in enhancing the accuracy of machine learning models. LightGBM, like other tree-based algorithms, offers a built-in way to look at feature importance, which can be a crucial aspect of machine learning for understanding which features are most influential in making predictions. LightGBM provides two types of feature importance: 1. Split-based feature importance: This method calculates the number of times a feature is used to split the data across all trees. The thought here is that if a feature is often selected for splitting, it must be because it's particularly useful for making predictions. 2. Gain-based feature importance: This method calculates the total gain of a feature when it's used in trees. In other words, it measures the total improvement in the model as a result of splits based on a particular feature. Gain-based importance considers how much a feature contributes to improving the model's prediction.

2.2.3 Monte Carlo Feature Selection (MCFS)

MCFS [3] is a feature selection method proposed by Szymon Dramiński in the context of machine learning and data analysis. It is designed to identify the most relevant features from a given dataset by evaluating their importance through a Monte Carlo simulation process. Here's a step-by-step description of the MCFS process: 1. Subsampling: MCFS starts by creating multiple random subsets of the original dataset, each containing a different combination of features. And for each subset of features, multiple random subsets of training data are constructed. 2. Classifier Training: For each subset of training data, a decision tree classifier is trained using only the selected features. The performance of the classifier is evaluated on a validation set or through cross-validation. 3. Importance Estimation: The importance of each feature is calculated based on weighted accuracy, information gain and coverage of the nodes. 4. Feature Ranking: After obtaining the feature importance scores, MCFS ranks the features based on their importance values. Features with higher importance scores are considered more relevant for the task at hand.

2.2.4 Random forests (RF)

RF [4] are a popular and powerful machine learning technique. They are an

ensemble learning method, meaning that they combine the predictions of several base estimators to improve generalizability and robustness over a single estimator. The base estimators in random forests are decision trees. Feature importance in random forests is a measure of how much each feature contributes to the predictions made by the model. It is calculated by looking at how much the tree nodes, which use a particular feature, reduce impurity on average (across all trees in the forest). Impurity is measured using Gini impurity or entropy, which are mathematical methods of quantifying disorder or uncertainty. Feature importance in random forests is calculated using the following process: 1. For each tree in the forest, find the total decrease in impurity from splitting on the feature. This is done by comparing the impurity of the parent node (before the split) with the weighted impurity of the child nodes (after the split). 2. Calculate the average decrease in impurity from splitting on the feature across all trees in the forest. 3. Normalize the results so that the sum of the importance of all features is equal to 1.

2.2.5 Categorical Boosting (CATboost)

CATboost [5] is a gradient boosting library developed by Yandex that is known for its exceptional performance in machine learning tasks. CATboost really shines when it comes to handling categorical features. It has an efficient and inbuilt method for processing categorical variables that outperforms traditional methods such as one-hot encoding. CATboost offers competitive performance and sometimes even outperforms other popular gradient boosting libraries like XGBoost and LightGBM. It provides highly accurate results, although at times, it may be slightly slower than its counterparts. Feature importance in CATboost quantifies the total contribution of each feature to the model prediction for a given dataset. CATboost calculates feature importance using Prediction Values Change. For each feature, it calculates the average change in the prediction value when this feature is excluded from the model. A larger change signifies higher importance.

2.2.6 Extreme Gradient Boosting (XGBoost)

XGBoost [6] is a highly popular open-source machine learning library. It's known for its speed, performance, and efficiency in handling a wide range of machine learning tasks, especially in structured or tabular data. XGBoost consistently delivers high-performance results and is often the go-to algorithm in winning Kaggle competition solutions. The algorithm uses gradient boosting framework at its core and is optimized for both system performance and machine learning performance. Here are three common ways feature importance can be calculated with XGBoost: 1. Weight: The number of times a feature appears in a tree across all the trees in the model. 2. Coverage: The number of times a feature is used to split the data across all trees, weighted by the number of training data points affected by the feature. A feature used to make splits on a large number of instances would have a higher coverage value. 3. Gain: The average contribution of a feature when it is used in trees. It is calculated as the total gain of each feature when it is used in all trees. Gain provides a measure of the improvement in accuracy brought by a feature to the branches it is on.

References

1. Ranstam, J.; Cook, J. Lasso regression. *Journal of British Surgery* **2018**, *105*, 1348-1348.
2. Ke, G.; Meng, Q.; Finley, T.; Wang, T.; Chen, W.; Ma, W.; Ye, Q.; Liu, T.-Y. Lightgbm: A highly efficient gradient boosting decision tree. *Advances in neural information processing systems* **2017**, *30*, 3146-3154.
3. Dramiński, M.; Koronacki, J. Rmcfs: An r package for monte carlo feature selection and

interdependency discovery. *Journal of Statistical Software* **2018**, *85*, 1 - 28.

4. Breiman, L. Random forests. *Machine learning* **2001**, *45*, 5-32.
5. Dorogush, A.V.; Ershov, V.; Gulin, A. *Catboost: Gradient boosting with categorical features support*. arXiv: 2018.
6. Chen, T.; Guestrin, C. In *Xgboost: A scalable tree boosting system*, Proceedings of the 22nd ACM SIGKDD International Conference on Knowledge Discovery Data Mining, 2016.
